# The upregulation of circFNDC3B aggravates the recurrence after endoscopic submucosal dissection (ESD) in early gastric cancer (EGC) patients

**DOI:** 10.1038/s41598-022-07154-y

**Published:** 2022-04-13

**Authors:** Jing Zhang, Jun Bai, Hongbing Zhu, Wei Li, Qunxing An, Dongxu Wang

**Affiliations:** 1 Department of Gastroenterology and Hepatology, Chinese PLA NO. 254 Hospital, 60 Huangwei Rd, Tianjin, 300142 China; 2 Department of Blood Transfusion, Xijing Hospital, Fourth Military Medical University, 127 Changle West Rd, Xi’anShaanxi Province, 710032 China

**Keywords:** Biochemistry, Cancer

## Abstract

It has been reported that the expression of CD44 variant 9 could be utilized as a predictive marker for the recurrence in early gastric cancer (EGC) after endoscopic submucosal dissection (ESD). And circFNDC3B was proved to increase the migration and invasion of gastric cancer (GC) cells. In this study, we recruited 96 EGC patients after ESD treatment and grouped them into High circFNDC3B expression group (High expression group) and Low circFNDC3B expression group (Low expression group). Accordingly, we found that the recurrence-free rate in the High expression group was lower than that in the Low expression group. In the High expression group, the relative expression of miR-942 and miR-510 was both suppressed while the relative expression of CDH1 mRNA and CD44 mRNA/protein was increased compared with those in the Low expression group. CircFNDC3B was found to target miR-942 and miR-510 and suppress their expressions respectively. Moreover, miR-942 was found to target CD44 mRNA while miR-510 was found to target CDH1 mRNA. The overexpression of circFNDC3B led to the down-regulation of miR-942 and miR-510, which accordingly resulted in the up-regulation of CD44 and CDH1 in MKN28 cells. Moreover, we found *H. pylori* infection could promote the expression of circFNDC3B, which also resulted in up-regulated CD44 and CDH1 mRNA level in rTip-α cultivated MKN28 cells. In summary, our study demonstrated that a higher level of circFNDC3B could lead to the increased expression of CD44 and CDH1 via modulating the signaling pathways of miR-942/CD44 and miR-510/CDH1 in EGC patients. And the up-regulation of CD44 and CDH1 would accordingly result in a higher recurrence rate of EGC patients treated by ESD.

## Introduction

Endoscopic submucosal dissection (ESD) is actually a typically utilized therapy for early gastric cancer (EGC), and the prognosis of ESD is usually good^1^. Having said that, EGC recurrence may still appear after the patient undergoes total resection, while previous studies have presented that about 15% of stomach cancer patients will develop multiple primary gastric carcinomas^2,3^. Thus, the ideal strategy to identify EGC recurrences is to find ideal biomarkers for predicting EGC reoccurrence. In addition, the eradication of *Helicobacter pylori* (*H. pylori*) was found to lower the rate of recurrence of multiple EGC in patients treated with ESD, indicating that the presence of *H. pylori* infection or associated inflammation can possibly determine the outcome of EGC recurrence^4,5^.

Circular RNA (circRNA), which is covalently joined at the 3’ and 5’ termini, has been actually identified as a type of endogenous non-coding RNAs that are ubiquitously present in eukaryotic cells, but some researchers have found that some circRNAs may be able to translate proteins^6,7^. CircRNAs are shown to be resistant to RNase digestion^8^. Existing data also suggests that circRNAs exert a key effect on controlling gene expression in cells^9,10^. Depending on the above results, circFNDC3B is actually synthesized by the type III domain‐containing protein 3B (FNDC3B) positioned on chromosome 3 in humans, and the dysregulation of circFNDC3B was shown to promote cell migration^11,12^.

MicroRNAs (MiRNAs) are a type of non-coding endogenous RNAs^13,14^. MiRNAs are actually associated with the control of numerous cellular processes like cell growth, cell cycle regulation, angiogenesis as well as metabolic processes^15^. On top of that, past researches have revealed that the abnormal expression of certain miRNAs can play an essential role in the growth, development, invasion as well as apoptosis of tumors^16^. Additionally, it was presented that the expression of miR-328 in the tissues positive for recurrence was greatly reduced compared with that in the tissues negative for recurrence, while the mRNA as well as protein expression of CD44 in the tissues positive for recurrence was obviously higher than that in the tissues negative for recurrence^17^.

The de novo germline mutation of CDH1, i.e., c. 1792 C [T (R598X)], was first found in a woman with a daughter diagnosed as early onset diffuse gastric cancer^18^. Alternatively, germline mutations of CDH1 germline were additionally found in some patients with early onset gastric cancer who are usually younger than 35 years upon diagnosis^19^. While the incidence of stomach cancer is pretty high in Japan and other Asian countries, the rate of germline mutation of CDH1 in Asian FGC subjects is lower than that in European subjects^20^.

It has been previously reported that the expression of CD44 variant 9 could be utilized as a predictive marker for the recurrence of EGC after ESD, and circular RNA FNDC3B was proved to increase the migration and invasion of GC cells^21,22^. A suppressed CDH1 protein level could promote the epithelial–mesenchymal transition (EMT) in GC, while the increased CD44 expression was also associated with cell adhesion^21^. In this study, we performed computational analysis to study the molecular relationships between circFNDC3B and CD44/CDH1 in the pathogenesis of EGC recurrence after ESD. And we also studied a group of EGC patients to analyze the association between the recurrence-free rate and the expression levels of circFNDC3B in these patients.

## Materials and methods

### Patient recruitment

In this study, a total of 96 EGC patients (TNM classification: T1A) who were subjected to ESD treatment were recruited and their expression of circFNDC3B was measured for grouping. Subsequently, the median circFNDC3B expression was calculated and utilized as the indicator to divide the patients into a Low expression group (N = 48, including all EGC patients whose expression level of circFNDC3B was at or above the median expression of circFNDC3B) and a High expression group (N = 48, including all EGC patients whose expression level of circFNDC3B was below the median expression of circFNDC3B). Therefore, for further analysis, since the patients were grouped according to their median, there are same number of patients in each group. The demographic and clinic parameters of both patient groups, including their sex, age, BMI, status of *H. pylori* infection, history of alcohol abuse and smoking, tumor site, as well as clinical grade of ESD was collected by reviewing their medical records, carrying out breath test for *H. pylori* infection, serological examinations, as well as bacterial culture, and the demographic and clinic parameters of the two patient groups were compared using Student’s test. In this study, ESD was defined as a type of adenocarcinoma constrained to the mucosa tissues or submucosa tissues in the stomach. All subjects with a past ESD history or those who received treatment for multiple ESD were not enrolled. An endoscopic forceps biopsy operation was carried out in each patient to collect ESD tissue samples for subsequent Western blot, qPCR and IHC assays. ESD follow-ups were carried out 2, 3, 6, 9, 12, 24, as well as 36 months after the initial ESD operation. The level of gastric atrophy was assessed using histological evaluation results based on the endoscopic atrophy border scale developed previously (Kimura et al. 1969; Ito et al. 1996; Satoh et al. 1996). Institutional ethical committee of Chinese PLA NO.254 Hospital has approved the protocol of this study. All methods were performed in accordance with the last vision of the Declaration of Helsinki. Written informed consent was obtained from all patients before the study.

### Cell culture and transfection

In this study, MKN28 cells, a extensively-studied gastric tubular adenocarcinoma cell line which were established from a 70-year-old female patient, were used to carry out cellular experiments. In brief, MKN28 cells were acquired from American Type Culture Collection (ATCC, Manassas, VA) and cultured according to the recommended conditions provided by the manufacturer, i.e., the cells were cultured in a Roswell Park Memorial Institute 1640 (RPMI 1640) medium (Gibco, Thermo Fisher Scientific, Waltham, MA) supplemented along with 10% of fetal bovine serum as well as 1% of penicillin and 100 U/ml of streptomycin. The culture conditions were 37 °C, 5% CO2 and saturated humidity. Furthermore, all cells were regularly examined to affirm the absence of Mycoplasma. When the cells reached 70% confluence, they were sub-cultured and divided into different groups. In cell model I, the MKN28 cells were divided into 2 groups, i.e., 1. pGL group (MKN28 cells transfected with an empty plasmid); and 2.pGL-FNDC3B group (MKN28 cells transfected with a pGL3 plasmid inserted with the circFNDC3B fragment). In cell model II, the MKN28 cells were also divided into 2 groups, i.e., 1. NC siRNA group (MKN28 cells transfected with a scramble negative control NC siRNA); and 2.pGL-FNDC3B group (MKN28 cells transfected with circFNDC3B siRNA to silence the expression of circFNDC3B). In cell model III, we utilized the MKN28 cells to establish a *H. pylori*-infected gastric cancer cell model in comparison with un-infected cell model. The MKN28 cell were established as 2 groups, i.e., 1. Control group (MKN28 cells in unprocessed medium); and 2. rTip-α group (MKN28 cells cultured in medium containing 12.5 μg/mL rTip-α).

According to protocols provided by a previous publication^22^, to obtain rTip-α, we transfected Tip-α into Escherichia coli for subsequent amplification. And the amplified rTip-α was purified for subsequent cell model establishment. All transfections were carried out using Lipofectamine 2000 (Invitrogen, Carlsbad, CA) according to the recommended transfection conditions provided by the manufacturer, and the transfected cells were harvested 48 h after the start of transfection to analyze the expression of target genes.

### RNA isolation as well as real-time PCR

The harvested cell as well as tissue samples were treated by utilizing a miRCURY RNA Isolation Kit (Exiqon, Qiagen, Germantown, MD) according to the recommended assay methods provided by the assay kit manufacturer to isolated cellular RNA. Then, the isolated RNA was assayed on an Agilent 2100 Bioanalyzer (Agilent Technologies, Mountain View, CA) in conjunction with an RNA 6000 Pico assay kit (Agilent Technologies, Mountain View, CA) according to the recommended assay methods provided by the assay kit manufacturer to quantify the RNA concentration. In the next step, 1 μg of isolated total RNA was converted into cDNA by making use of a QuantiTect Reverse Transcription assay kit (Qiagen, Germantown, MD) according to the recommended assay methods provided by the assay kit manufacturer. Finally, real time quantitative polymerase chain reaction (RT-qPCR) was performed on a BX-384 real time PCR apparatus (Bio-Rad laboratories, Hercules, CA) by making use of a QuantiTect SYBR Green PCR assay kit (Qiagen, Germantown, MD) according to the recommended assay methods provided by the assay kit manufacturer to evaluate the relative expression of circFNDC2B, miR-942, miR-510, CD44 mRNA as well as CDH1 mRNA in each sample using the 2^−ΔΔCt^ approach. The expression of GAPDH in each sample was used as the internal control.

### Vector construction, mutagenesis, and luciferase assay

We utilized online bioinformatic tools including TargetScan (http://www.targetscan.org/vert_80/) and miRDB (http://mirdb.org/) to compare the sequences of circFNDC2B, miR-942, miR-510, CD44 mRNA and CDH1 mRNA. Accordingly, we detected a putative binding site of miR-942 on circFNDC3B, while a putative binding site of miR-942 was detected on the 3’UTR of CD44 mRNA. Similarly, we detected a putative binding site of miR-510 on circFNDC3B, while a putative binding site of miR-510 was detected on the 3’UTR of CDH1 mRNA. In the next step, we performed luciferase assays in MKN28 cells to confirm the regulatory relationship of circFNDC2B/miR-942, circFNDC2B/miR-510, miR-942/CD44 mRNA, and miR-510/CDH1 mRNA. In brief, the wild type sequences of circFNDC2B, CD44 mRNA, and CDH1 mRNA containing the corresponding miRNA binding sites were cloned into pGL plasmid vectors to generate wild type plasmids of circFNDC2B, CD44 mRNA, and CDH1 mRNA. At the same time, the sequences of circFNDC2B, CD44 mRNA, and CDH1 mRNA containing the corresponding miRNA binding sites were subject to site-directed mutagenesis to generate mutant type sequences of circFNDC2B, CD44 mRNA, and CDH1 mRNA containing the corresponding miRNA binding sites, which were also cloned into pGL plasmid vectors to generate mutant type plasmids of circFNDC2B, CD44 mRNA, and CDH1 mRNA. In the next step, MKN28 cells were co-transfected with the plasmids carrying wild type or mutant type circFNDC2B, CD44 mRNA, and CDH1 mRNA along with miR-510 and miR-942. At 48 h post transfection, the luciferase activity of transfected cells was assayed by utilizing a GloMax Multi Detection assay kit (Promega, Madison, WI) according to the recommended assay methods provided by the assay kit manufacturer.

### Recurrence-free rate

The recurrence-free rate of the patients was analyzed by using R statistical software (version 3.0). The recurrence-free rate in each group was calculated at various follow-up time points, and the recurrence-free rates of the two groups were compared at the level of statistical significance of 0.05. The Kaplan–Meier survival curves were generated for high and low groups of circFNDC3B expression.

### Western blot analysis

Cells as well as tissue samples were first lysed in a phosphatase- and protease-inhibitor containing 1X RIPA buffer (Thermo Fisher Scientific, Waltham, MA) according to the recommended assay methods provided by the assay kit manufacturer. The collected lysates were then centrifuged to collect proteins in the supernatant, whose concentration of total proteins was examined by using a BCA protein assay kit (Pierce, Thermo Fisher Scientific, Waltham, MA) according to the recommended assay methods provided by the assay kit manufacturer. In the next step, the protein in each sample was resolved by 10% SDS-PAGE and blotted onto nitrocellulose membranes (Hybond, GE Medical Care, Pittsburgh, PA), which was blocked with TBSS containing 5% of skim milk and subsequently incubated with primary anti-CD44 and anti-CDH1 antibodies as well as HRP-conjugated secondary antibodies in sequence according to the recommended antibody incubation conditions provided by the assay kit manufacturer (Abcam, Cambridge, CA). Finally, after the protein blots (all original protein blots are shown in Supplementary file) were developed by using an enhanced chemiluminescence Western blot substrate (Pierce, Rockford, IL) according to the recommended assay methods provided by the assay kit manufacturer, the relative protein expression of CD44 and CDH1 in each sample was calculated.

### IHC assay

Collected tissue samples were paraffin embedded, sliced into 4 um sections, deparaffinized, gradient alcohol hydrated, and incubated with primary anti-CD44 antibodies and biotin-labeled secondary antibodies in sequence according to the recommended antibody incubation conditions provided by the assay kit manufacturer (Abcam, Cambridge, CA) to determine the positive protein expression of CD44 in each sample under a Zeiss Axioskop microscope.

### Statistical analysis

Unless otherwise specified, all results are presented as mean ± S.E.M of 4 independent tests. Statistical evaluations were done by making use of the Student's t test in SPSS 21.0 software (IBM, Chicago, IL) and Prism 8.0 program (GraphPad, San Diego, CA), and *P* < 0.05 was deemed as statistically significant.

## Results

### EGC patient recruitment

In this study, a total of 96 EGC patients who were subjected to ESD treatment were recruited. Their expression of circFNDC3B was measured, and the median circFNDC3B expression was calculated and utilized as the indicator to group the patients into a Low expression group (N = 48) and a High expression group (N = 48). The demographic and clinic parameters of both patient groups were collected and recorded. As shown in Table 1, we did not detect any significant differences in respect to the recorded parameters between the Low group and the High group.

**Table 1:** Demographic and clinical parameters of EGC patients subjected to ESD treatment.

Characteristics	Low expression (N = 48)	High expression (N = 48)	*P* value
Age, years	65.0 ± 5.6	60.0 ± 6.4	0.555
**Gender**			
Female	33	38	0.614
Male	15	10	
BMI (kg/m^2^)	24.9 ± 2.9	25.0 ± 3.4	0.644
number of *H. pylori*	18 (37.5)	15 (33.1)	0.514
**Drinking history**			
Ever	23	22	0.8845
Never	21	21	
No record	4	5	
**Smoking history**			
Ever	18	20	0.1979
Never	24	25	
No record	6	3	
**Differentiation**			
Differentiated	42	44	0.2963
UNDifferentiated	6	4	
**ULCER**			
Negative	39	40	0.6985
Positive	9	8	

### Expression of candidate genes in EGC patient groups

**Figure 1:**
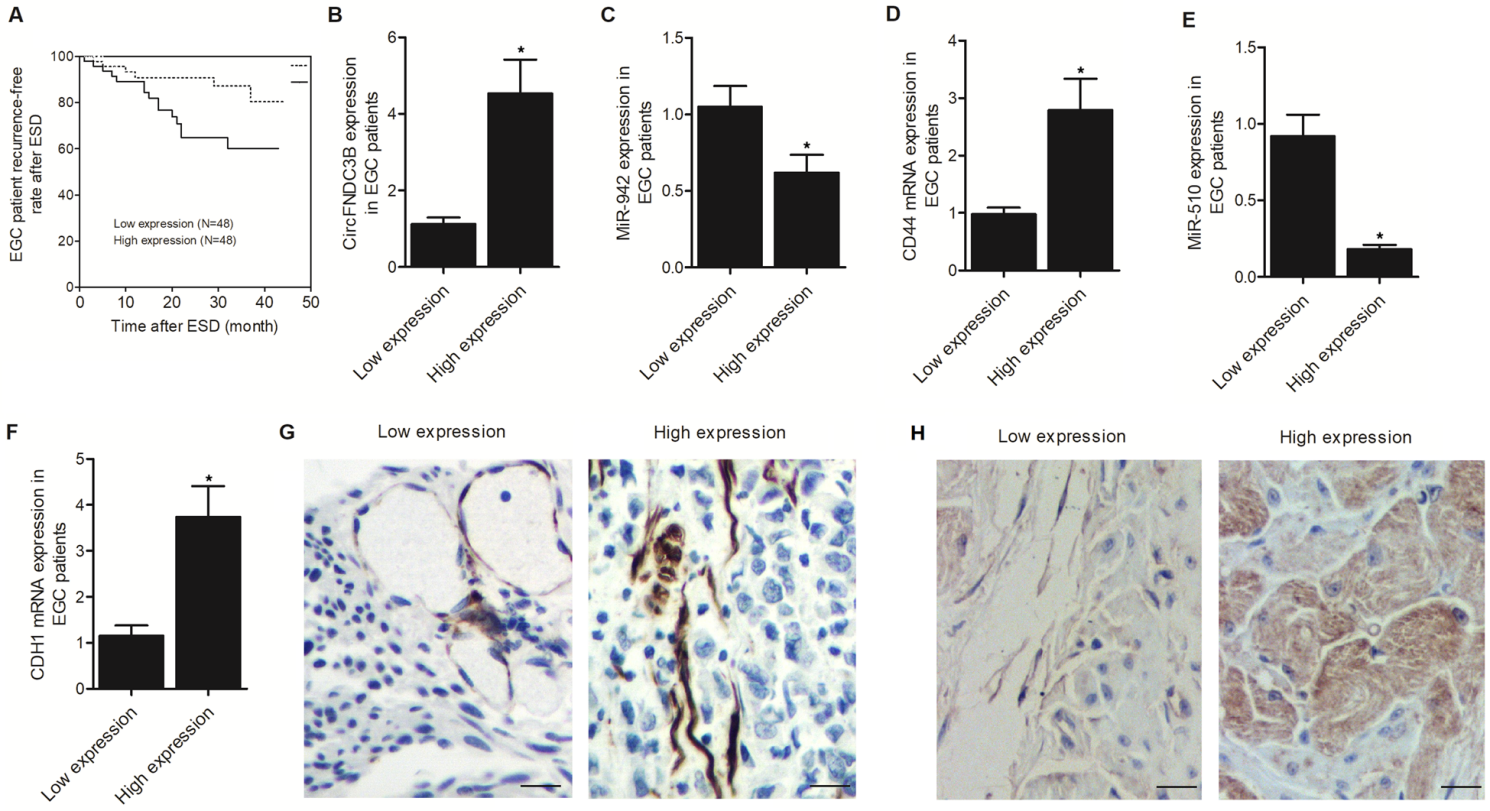
Expression of candidate genes in EGC patient groups (*P value < 0.05 vs. Low expression group). (**A**) The recurrence-free rate was lower in the High expression group (P value = 0.0174); (**B**) CircFNDC3B expression was higher in the High expression group; (**C**) MiR-942 expression was reduced in the High expression group; (**D**) CD44 mRNA expression was increased in the High expression group; (**E**) MiR-510 expression was decreased in the High expression group; (**F**) CHD1 mRNA expression was elevated in the High expression group; (**G**) IHC indicated higher CD44 expression in the High expression group (scale bar: 100 μm); (**H**) IHC indicated higher CHD1 expression in the High expression group (scale bar: 100 μm).

The recurrence-free rate was calculated and plotted to study the potential relationship between circFNDC3B expression and the risk of recurrence. As shown in Fig. 1A, the recurrence-free rate in the High expression group was lower than that in the Low expression group. Therefore, it is suspected that the higher circFNDC3B expression is correlated with a higher rate of recurrence after ESD in EGC patients. All recruited EGC patients were grouped according to their circFNDC3B expression levels (Fig. 1B). Accordingly, in the High expression group that exhibited a relatively higher level of circFNDC3B, the relative expression of miR-942 (Fig. 1C) and miR-510 (Fig. 1E) was both suppressed. Also, the relative expression of CD44 mRNA (Fig. 1D) and CDH1 mRNA (Fig. 1F) was elevated in the High expression group. Moreover, IHC assay also showed increased CD44 expression (Fig. 1G) and CDH1 expression (Fig. 1H) in the High expression group compared with that in the Low expression group.

### Establishment of signaling pathways

**Figure 2:**
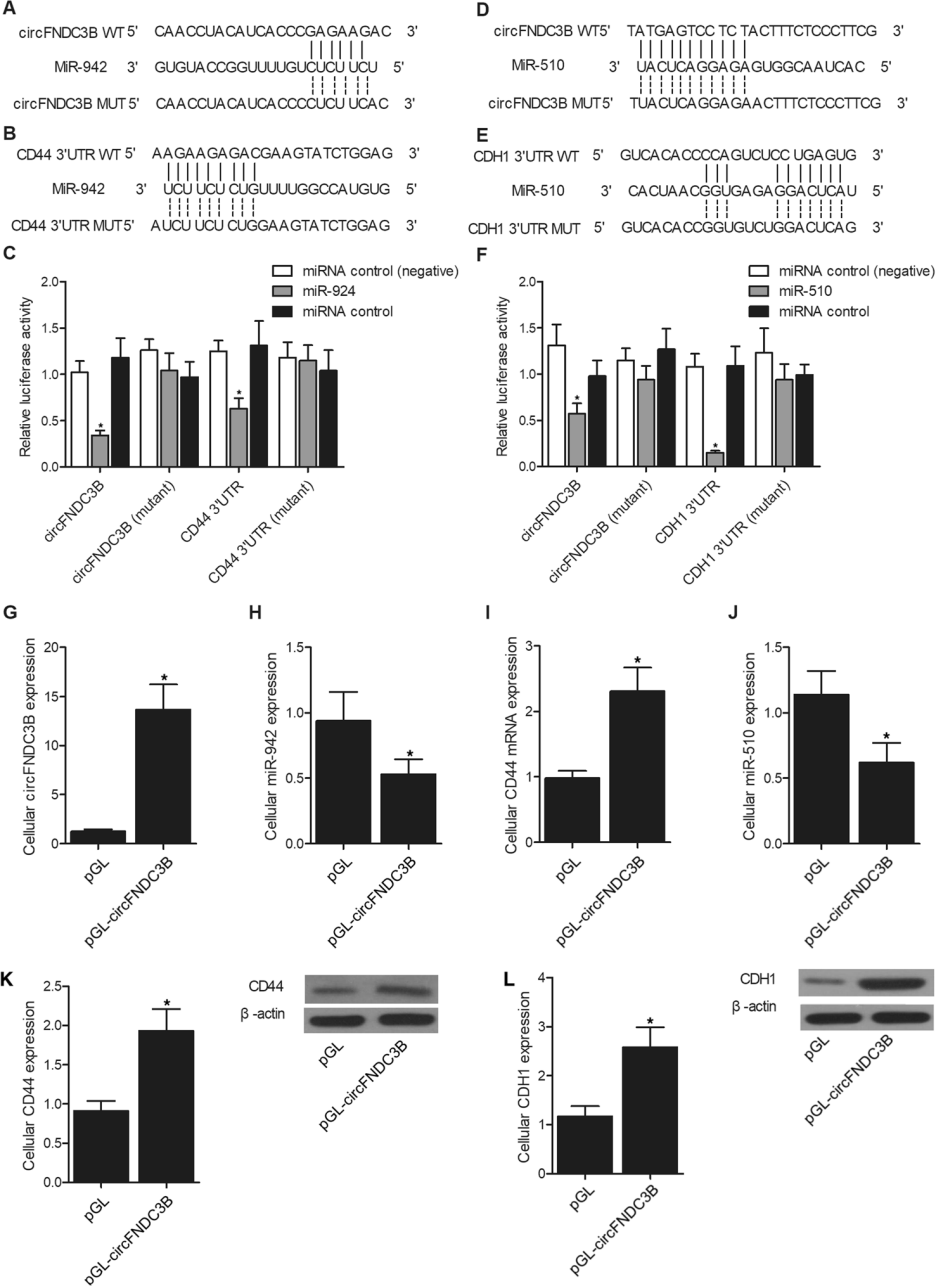
Signaling pathway establishment. (**A**) Sequence comparison between circFNDC2B and miR-942; (**B**) Sequence comparison between CD44 mRNA and miR-942; (**C**) Luciferase activity of wild type circFNDC2B and wild type CD44 mRNA was both evidently reduced in the presence of miR-942 (*P value < 0.05 vs. miRNA control (negative) group); (**D**) Sequence comparison between circFNDC2B and miR-510; (**E**) Sequence comparison between CHD1 mRNA and miR-510; (**F**) Luciferase activity of wild type circFNDC2B and wild type CHD1 mRNA was both evidently reduced in the presence of miR-942 (*P value < 0.05 vs. miRNA control (negative) group); (**G**) CircFNDC3B expression was elevated in MKN28 cells overexpressing circFNDC3B (*P value < 0.05 vs. pGL group); (**H**) MiR-942 expression was inhibited in MKN28 cells overexpressing circFNDC3B (*P value < 0.05 vs. pGL group); (**I**) CD44 mRNA expression was promoted in MKN28 cells overexpressing circFNDC3B (*P value < 0.05 vs. pGL group); (**J**) MiR-510 expression was up-regulated in MKN28 cells overexpressing circFNDC3B (*P value < 0.05 vs. pGL group); (**K**) CD44 expression was higher in MKN28 cells overexpressing circFNDC3B (*P value < 0.05 vs. pGL group); (**L**) CDH1 expression was higher in MKN28 cells overexpressing circFNDC3B (*P value < 0.05 vs. pGL group).

**Figure 3:**
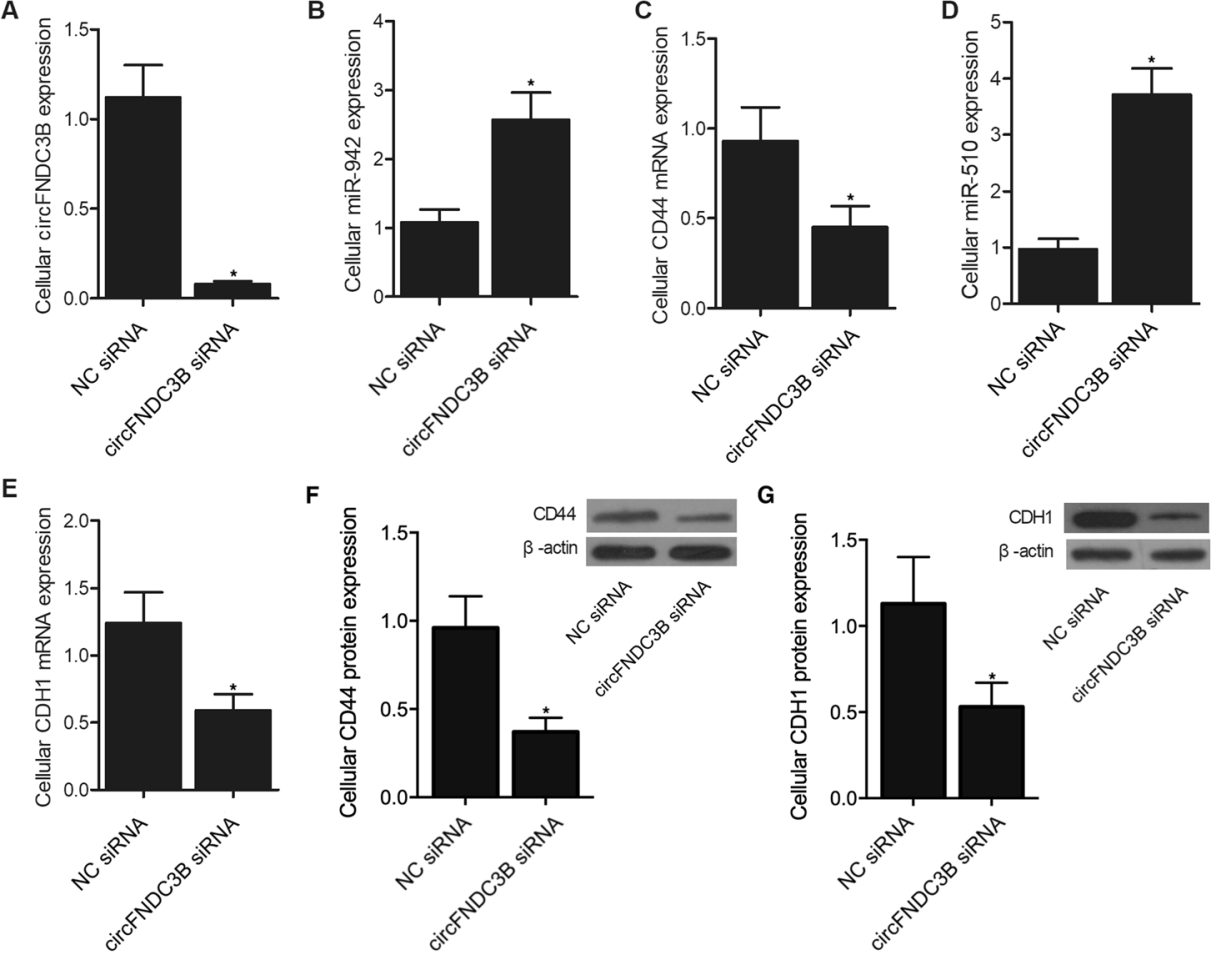
Validation of signaling pathway establishment (*P value < 0.05 vs. NC siRNA group). (**A**) CircFNDC3B expression was inhibited by the knockdown of circFNDC3B in MKN28 cells; (**B**) MiR-942 expression was up-regulated by the knockdown of circFNDC3B in MKN28 cells; (**C**) CD44 mRNA expression was suppressed by the knockdown of circFNDC3B in MKN28 cells; (**D**) MiR-510 expression was increased by the knockdown of circFNDC3B in MKN28 cells; (**E**) CDH1 mRNA expression was down-regulated by the knockdown of circFNDC3B in MKN28 cells; (**F**) CD44 protein expression was suppressed by the knockdown of circFNDC3B in MKN28 cells; (**G**) CDH1 protein expression was down-regulated by the knockdown of circFNDC3B in MKN28 cells.

We utilized online bioinformatic tools to compare the sequences of circFNDC2B, miR-942, miR-510, CD44 mRNA and CDH1 mRNA. Accordingly, we detected a putative binding site of miR-942 on circFNDC3B (Fig. 2A), while a putative binding site of miR-942 was detected on the 3’UTR of CD44 mRNA (Fig. 2B). We further performed luciferase assay in MKN28 cells to establish the circFNDC3B/miR-942/CD44 signaling pathway. As shown in Fig. 2C, MKN28 cells were transfected with plasmids carrying wild type or mutant circFNDC3B along with the transfection of miR-924 or miRNA positive and negative controls. Accordingly, the luciferase activity was significantly reduced in MKN28 cells co-transfected with wild type circFNDC3B and miR-924, indicating circFNDC3B could sponge miR-924. Also, in MKN28 cells transfected with wild type or mutant 3’UTR of CD44 mRNA, the luciferase activity was only suppressed in the presence of miR-924 and wild type CD44 3’UTR, indicating CD44 mRNA was targeted by miR-942. Moreover, we also detected two binding sites of miR-510 in circFNDC3B (Fig. 2D) and 3’UTR of CDH1 mRNA (Fig. 2E), respectively, and the luciferase activity assay (Fig. 2F) indicated that the transfection of miR-510 inhibited the luciferase activity of wild type circFNDC3B in MKN28 cells, while the luciferase activity of wild-type CDH1 3’UTR in MKN28 cells was also significantly reduced by the transfection of miR-510, thus establishing a circFNDC3B/miR-510/CDH1 signaling pathway.

### Overexpression of circFNDC3B regulated signaling intensity

To further validate the above established signaling pathways, we transfected PENCTE01 cells with plasmids carrying circFNDC3B or empty plasmids. Accordingly, the success of transfection was validated by the sharply increased cellular circFNDC3B expression in the pGL-FNDC3B group (Fig. 2G). And the expression of miRNAs including miR-942 (Fig. 2H) and miR-510 (Fig. 2J) was inversely regulated by the overexpression of circFNDC3B in MKN28 cells. Moreover, the expression of CD44 mRNA (Fig. 2I) was also markedly increased by the transfection of plasmids carrying circFNDC3B, and the trend was further validated by the significantly increased protein expression of CD44 (Fig. 2K) and CDH1 (Fig. 2L).

### Knockdown of circFNDC3B regulated signaling intensity

MKN28 cells were also transfected with circFNDC3B siRNA or a negative control siRNA, respectively. Accordingly, the transfection of circFNDC3B siRNA resulted in the successful knockdown of circFNDC3B in MKN28 cells (Fig. 3A), and the expression of miRNAs including miR-942 (Fig. 3B) and miR-510 (Fig. 3D) was evidently increased by the knockdown of circFNDC3B. On the other hand, the cellular expression of CD44 mRNA (Fig. 3C) and protein (Fig. 3F), as well as CDH1 mRNA (Fig. 3E) and protein (Fig. 3G), were inhibited by the knockdown of circFNDC3B. Therefore, it can be concluded that the level of circFNDC3B can be used as a biomarker to predict the risk of EGC recurrence after ESD.

### Infection of *H. pylori* regulated signaling intensity

**Figure 4:**
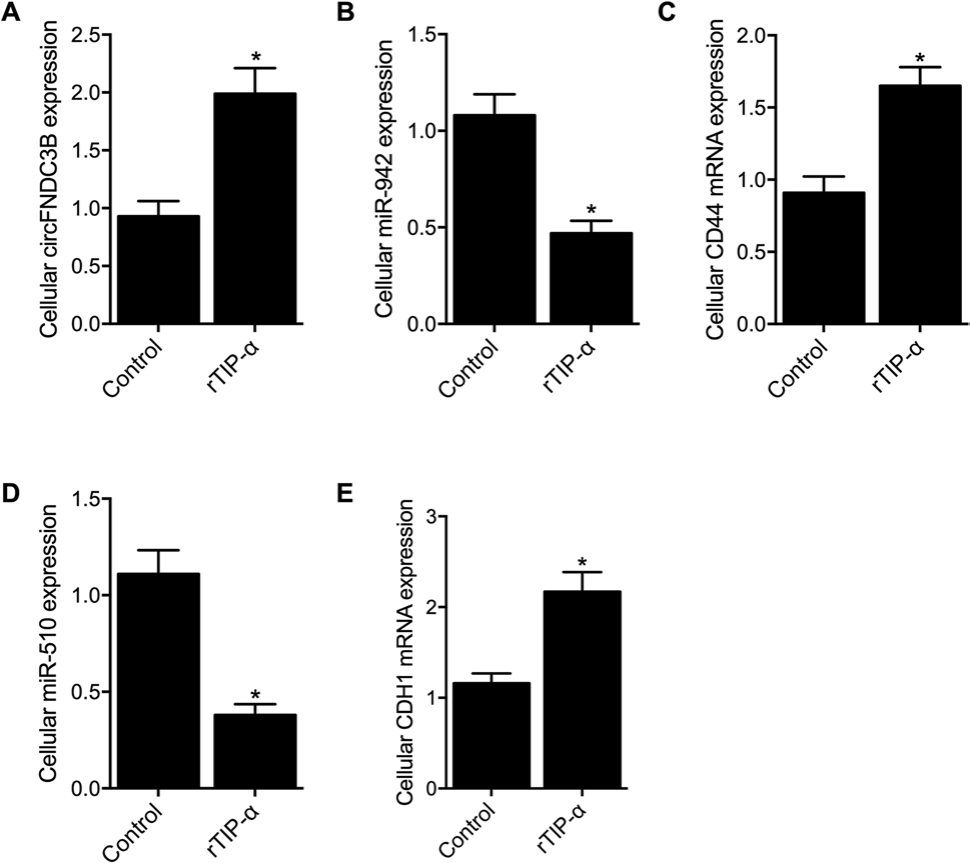
Influence of *H. pylori* infection upon the signaling pathway (*P value < 0.05 vs. control group). (**A**) CircFNDC3B expression was increased in the rTip-α group compared with the control group; (**B**) The level of miR-942 was suppressed in the rTip-α group compared with the control group; (**C**) Expression of CD44 mRNA was higher in the rTip-α group compared with the control group; (**D**) The level of miR-510 was suppressed in the rTip-α group compared with the control group; (**E**) Expression of CDH1 mRNA was higher in the rTip-α group compared with the control group.

MKN28 cells were cultured with rTip-α to create a *H. pylori*-infected cell model. Compared with the Control group, the expression of circFNDC3B was increased in the rTip-α group (Fig. 4A). Accordingly, both the cellular miR-942 level (Fig. 4B) and miR-510 level (Fig. 4D) were significantly decreased in the rTip-α group, and the cellular gene expression of CD44 mRNA (Fig. 4C) and CDH1 mRNA (Fig. 4E) was evidently up-regulated.

## Discussion

In this study, we collected samples from the EGC patients receiving ESD treatment. Those samples were divided into two groups based on their expression level of circFNDC3B (high expression group vs. low expression group). The recurrence-free rate in the High expression group was lower than that in the Low expression group. In the High expression group, the relative expression of miR-942 and miR-510 was both suppressed, while the relative expression of CDH1 and CD44 was increased compared with those in the Low expression group.

In a past study, it was found that circFNDC3B lowered the expression of E-cadherin while improving the expression of CD44, thus promoting the migration as well as invasion of GC cells. From this point of view, if circFNDC3B expression is dysregulated, it is actually closely correlated with the severity of malignancy as well as highlighting the properties of cell invasion and migration. Thus, the ectopic circFNDC3B expression could be used as a biomarker for predicting the distant metastasis of GC cells^21^. MiR-942 was actually found to modulate the progression of colon cancer, esophageal squamous cell carcinoma, as well as ovarian cancer^12–14^. The role of miR-942 in regulating the biological features of BCa cells was actually extensively studied. The results of such studies revealed that the over-expression of miR-942 considerably promoted the viability of cells while reducing cell apoptosis. In this study, by using computational analysis and luciferase assays, we found that circFNDC3B could sponge the expression of miR-942 and miR-510, respectively, while miR-942 and miR-510 targeted CD44 mRNA and CDH1 mRNA, respectively. In addition, we found that the overexpression of circFNDC3B led to the down-regulation of miRNAs including miR-942 and miR-510, while up-regulating the expression of CD44 and CDH1 in MKN28 cells. Similarly, the knockdown of circFNDC3B promoted the expression of miR-942 and miR-510 while downregulating the expression of CD44 and CDH1.

A past research showed that the expression of CD44v9, a marker of stem-like cancer cells, in EGC can be used to precisely forecast the relapse of EGC after the ESD treatment, indicating that post-ESD follow-ups need to be performed much more frequently in individuals carrying tumors positive for the expression of CD44v9^23^. Yet CD44 is another molecule that might affect the carcinogenesis of EGC jointly with H pyloriis infection. As a type of adhesion molecules expressed on cell surface, CD44 has been found in many types of cells such as epithelial cells in the stomach. In addition, the cells showing positive CD44 expression have been demonstrated to have features similar to those of cancer stem cells, which were shown to have the potential to trigger the growth of tumors^24^. Moreover, GC cells positive for the expression of CD44 revealed self-renewal properties, while the knockdown of the expression of CD44 by siRNA lowered the colony formation ability of the tumors in mice that were immunodeficient^24^. The gastric cancer tissues showing signs of lymph node metastasis showed downregulated levels of miR-510, miR-24-1 as well as miR-1284, but the level of miR-10a expression was actually upregulated. As a result, it was shown that miR-10a expression is linked to the presence of lymph node metastasis but is independent of lymphatic invasion in subjects suffering from primary GC^25^. As a type of membrane protein whose activity is dependent on the level of calcium, E-cadherin s closely involved in the adhesion of cells to induce cell polarity^26^. The mutations in the CDH1 gene are often related to the increased risk of early onset diffuse gastric cancer^27^. The germline mutations in the genes of E-cadherin and CDH1 were actually recognized in people with the predisposition to diffuse GC. In fact, the GC risk in male carriers of CDH1 mutations who are older than 80 years of age can be up to 70%, while the GC risk in female carriers of CDH1 mutations who are older than 80 years of age can be up to 56%. So far, the primary gene linked to hereditary gastric cancer syndrome (HDGC) is CDH1, and the germline mutations of CDH1 have been linked to about 3% of all GC cases^28^.

However, the results obtained from our study was limited. The sample size was relatively small in this study, and the number and quantity of samples collected from the participants deterred us from more experiments which were necessary to draw a solid conclusion from our investigation. Further studies with larger sample size and more comprehensive analysis were warranted to confirm the result of this study.

## Conclusion

In summary, our study demonstrated that a higher level of circFNDC3B could lead to the increased expression of CD44 and CDH1 via modulating the signaling pathways of miR-942/CD44 and miR-510/CDH1 in EGC patients, and the upregulation of CD44 and CDH1 would result in a higher recurrence rate in EGC patients treated by ESD.

## Supplementary Information

Supplementary Information.

## Data Availability

The data that support the findings of this study are available from the corresponding author upon reasonable request.

## Abbreviations

ESD: Endoscopic submucosal dissection
EGC: Early gastric cancer
