# Supplementary material for: The upregulation of circFNDC3B aggravates the recurrence after endoscopic submucosal dissection (ESD) in early gastric cancer (EGC) patients
Source: Sci Rep. 2022 Apr 13;12:6178. doi: 10.1038/s41598-022-07154-y (PMC9007947; doi:10.1038/s41598-022-07154-y)

The upregulation of circFNDC3B aggravates the recurrence after endoscopic submucosal dissection (ESD) in early gastric cancer (EGC) patients

Jing Zhang[#1], Jun Bai[#1], Hongbing Zhu[1], Wei Li[1], Qunxing An*[2], Dongxu Wang*[1]

1. Department of Gastroenterology and Hepatology, Chinese PLA NO.254 Hospital, Tianjin, China, 300142
2. Department of Blood Transfusion, Xijing Hospital, Fourth Military Medical University, Xi'an, Shaanxi Province, China, 710032

*Corresponding author:

Dongxu Wang

Affiliation: Department of Gastroenterology and Hepatology, Chinese PLA NO.254 Hospital

Address: 60 Huangwei Rd, Tianjin, China, 300142

Email: lncRNAstudy@163.com

and

Qunxing An

Affiliation: Department of Blood Transfusion, Xijing Hospital, Fourth Military Medical University

Address: 127 Changle West Rd, Xi'an, Shaanxi Province, China, 710032

Email: skki88@163.com

[#]Authors contributed equally to this study

Western Blot Original Images

Original Image for Fig2K CD44

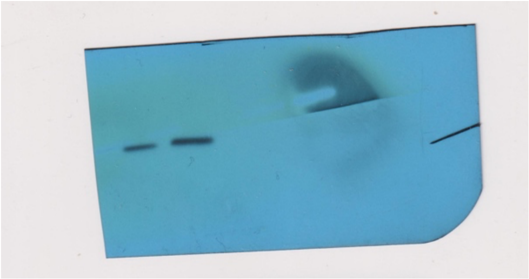

Original Image for Fig2K beta actin

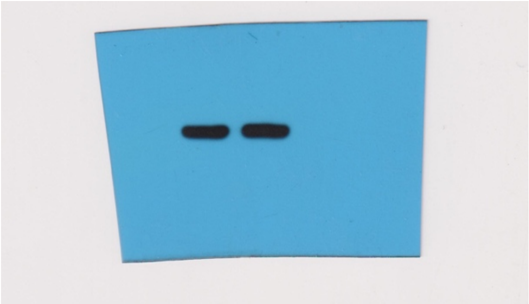

Original Image for Fig2L CDH1

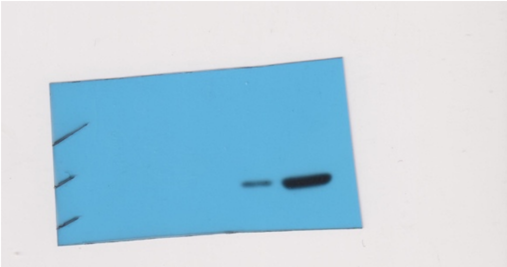

Original Image for Fig2L beta actin

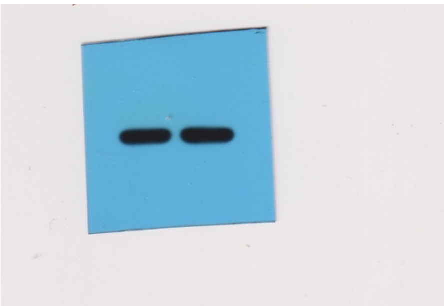

Original Image for Fig3F CD44

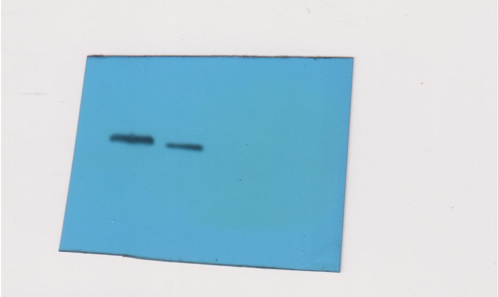

Original Image for Fig3F beta actin

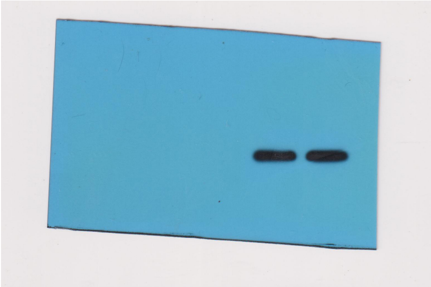

Original Image for Fig3G CDH1

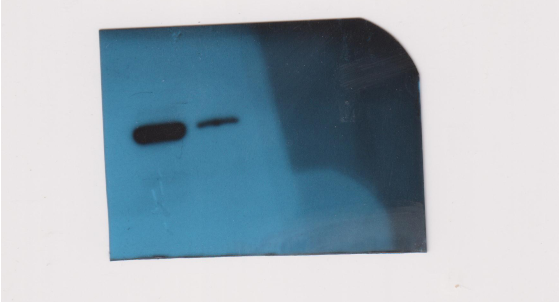

Original Image for Fig3G beta actin

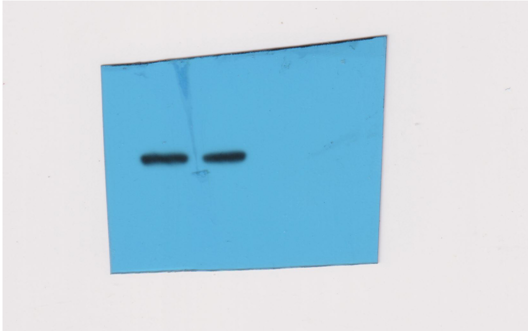

Supplement: Supplementary file 1 — Supplementary Information. [file 41598_2022_7154_MOESM1_ESM.pdf]
